# Implementing Better Standard Instruments for Detecting Cirrhosis in Hepatitis C Patients

**DOI:** 10.7759/cureus.44597

**Published:** 2023-09-03

**Authors:** Mark D Fagala, Yakov Shpak

**Affiliations:** 1 Clinical Research, Saba University School of Medicine, The Bottom, BES; 2 Clinical Research, Staten Island University Hospital/Zucker School of Medicine Hofstra/Northwell, Staten Island, USA

**Keywords:** fibrosis, children, adults, cirrhosis, hepatic venous portal gradient, fibroscan, transient elastography, liver biopsy, liver stiffness, hepatitis c

## Abstract

An increasing number of hepatitis C diagnoses in the younger population could partly be due to the rising opioid epidemic and intravenous drug use. Using hepatic venous portal gradient (HVPG) and liver stiffness tests, this study investigates better early diagnostic markers in identifying cirrhosis-related complications compared to percutaneous liver biopsy in hepatitis C patients. Scholarly journal articles were surveyed using PubMed and MeSH terms. Articles published more than 15 years ago were excluded. Various databases from the New England Journal of Medicine and the Centers for Disease Control and Prevention were also referenced to support the hypothesis. There is substantial affirmation from cohort and clinical studies that transient elastography and HVPG can indicate advancing chronic inflammatory and fibrotic stages of cirrhosis in comparison to liver biopsy. Additionally, they are helpful in predicting overall mortality from complications such as esophageal varices. The use of liver stiffness measurements and HVPG appears to be equivalent and/or superior to liver biopsy in assessing advancing cirrhosis. As hepatitis C cases continue to rise, it is crucial to search for alternative methods to better suit the needs of the patients and to improve their overall prognosis and potential treatments. Liver biopsy as the gold standard for cirrhosis assessment is questionable when less invasive instrumental tools are available in practice that have been shown to predict progressing fibrosis.

## Introduction and background

The Centers for Disease Control and Prevention (CDC) recently reported a sharp increase in cases of hepatitis C virus (HCV) due to the uncontrolled opioid crisis in the United States [1]. According to the CDC, “more than 3.5 million Americans are living with HCV, most being baby boomers born between 1945 and 1965 with recent incidences being seen in younger people addicted to opioids.” Between 2010 and 2015, HCV incidence rates increased by 167%, from 0.3 cases to 0.8 cases per 100,000 US population, with the highest primary risk factor among people who inject drugs [2]. The majority of these cases are seen in the Eastern United States, demonstrating a correlation with increased hospital and treatment program admissions for opioid use [2].

HCV belongs to a family of positive-sense, enveloped RNA viruses called Flavivirade and is the leading cause of liver transplants in the United States. Moreover, long-term infection with HCV can progress to a chronic stage in roughly 85% of patients, with cirrhosis in approximately 20% of patients and an increased risk of developing hepatocellular carcinoma (2018) [1]. The aim of this research is to determine the best methods to diagnose cirrhosis early, to guide management in slowing the disease process, and the complications associated with the chronic state. The current gold standard for assessing advanced stages of cirrhosis and liver fibrosis is invasive percutaneous liver biopsy. The aim of advancements in medicine is to use minimally or non-invasive techniques that will better assess these chronic states with supportive epidemiological data.

According to the New England Journal of Medicine, by 2040, deaths from chronic hepatitis are projected to exceed the combined mortality associated with HIV, tuberculosis, and malaria. In 2016, the World Health Organization called for a global strategy to eliminate viral hepatitis as a public health threat by 2030. Reliable epidemiological data are not available for hepatitis outcomes and related deaths are difficult to estimate. The aim of this study is to assess whether using hepatic venous portal gradient (HVPG) and liver stiffness tests are better early diagnostic markers in identifying cirrhosis-related complications compared to the current gold standard of percutaneous liver biopsy in hepatitis C patients. The stated hypothesis will demonstrate that using HVPG and liver stiffness tests are better early diagnostic markers in identifying cirrhosis-related complications compared to percutaneous liver biopsy in hepatitis C patients.

In the clinical setting, chronic liver diseases are evaluated based on viral and non-viral causes of cirrhosis. A wide variety of tests are used to systematically stage hepatic disease or cirrhosis to determine prognosis in individual patients and overall mortality due to complications. According to the Baveno VI consensus workshop, clinically significant portal hypertension is predictive of gastroesophageal varices and decompensation, which requires the prophylactic use of beta-blockers and endoscopic therapy (2008) [3]. Decompensated cirrhosis complications such as ruptured esophageal varices, portal hypertension, hepatorenal syndrome, and bacterial peritonitis are among the most common causes of mortality in patients with cirrhosis. In decompensated cirrhosis, the liver loses the ability to maintain its function due to severe damage and scarring which results in the observed pathophysiology. Compensated cirrhosis can still function normally; however, scarring from fibrosis is present with mild-to-moderate damage.

The significance of this study is to determine which screening tools are best at predicting mortality, early cirrhosis changes, and elevations in stiffness to catch the cases early enough for management.

Currently, the gold standard for measuring portal hypertension is the HVPG test. Patients are placed in a supine position and given lidocaine anesthetic. A balloon catheter is attached to a pressure transducer, inserted at the level of the internal jugular vein, and threaded to the hepatic vein under guided direction with intravenous (IV) contrast. A measurement is generated from the difference between the free hepatic venous pressure and the wedged hepatic venous pressure and is recorded as HVPG. In a healthy patient, this value is typically <5 mmHg. In patients with viral and non-viral causes of cirrhosis, this increases to >10 mmHg, which is deemed clinically significant portal hypertension (CSPH). Severe portal hypertension occurs in pressures exceeding >12 mmHg with decompensated complications such as ascites and variceal bleeding.

The basic principle of transient elastography (TE) is using a fibroscan device equipped with a probe including an ultrasonic transducer and sending a mild amplitude and low-frequency vibration wave propagating through tissue. This wave is measured based on its velocity through the tissue, which is directly related to liver stiffness, expressed as kilopascals. Thus, the harder or more fibrotic the tissue, the faster the shear wave progresses. During the procedure, patients are placed in the dorsal decubitus position with the right arm in maximal abduction. The probe is placed externally on the skin in the intercostal space on the right side at the level of the liver. The entire examination is non-invasive and takes less than five minutes. This is especially convenient as it is a quick and painless patient experience.

In patients who undergo percutaneous liver biopsy, a needle is placed through the skin of the right upper abdomen between the ribs at the level of the liver. Patients are placed under general anesthesia and are asleep for the procedure. The biopsy sample from the liver is taken and reviewed under the microscope for evaluation. Complications include bleeding, pain around the injection site, infection, gallbladder puncture with subsequent bile leakage, and pneumothorax or hemothorax.

To compare the efficacy of all, several tests have been implemented to measure disease severity and risk of adverse outcomes. For instance, the Child-Turcotte-Pugh (CTP) and Model for End-Stage Liver Disease (MELD) are tools implemented for determining mortality associated with adverse cirrhosis outcomes and worsening parameters such as ascites and esophageal varices. In the CTP score, there are five clinical measures for liver disease with each marker scored from 1 to 3. The clinical measures include total bilirubin, serum albumin, prothrombin time (PT)/international normalized ratio (INR), ascites, and hepatic encephalopathy. As more points are accumulated, survival is reduced and mortality risk increases, as indicated by the three classes of A, B, and C. For instance, class A has a better overall prognosis than class C. The MELD is a 90-day survival assessment scoring system for chronic liver disease severity and mortality. In much of the same methods used by the CTP, a patient’s bilirubin, serum creatinine, and INR are used to determine survival.

The Meta-Analysis of Histological Data in Viral Hepatitis (METAVIR) is a scoring system used to evaluate the severity of fibrosis from a liver biopsy sample in a hepatitis C patient. In other words, it determines the scarring and inflammation present in a sample. Additionally, it measures how fast the fibrosis is progressing which guides physicians on treatment and prognosis.

This study was conducted to assess the usefulness and accuracy of non-invasive methods in the management of liver disease in chronic HCV infection, including the detection and prognostication of cirrhosis and its complications, as well as response to treatment.

## Review

Methodology

Search Strategies

Databases used included PubMed (www.pubmed.org), New England Journal of Medicine (www.nejm.org), EBSCO host, Dynamed, Centers for Disease Control and Prevention (www.CDC.gov), and Google Scholar. PubMed was used primarily for the primary search of articles. The following search strategy was utilized: [cirrhosis] AND [liver stiffness] AND [Hepatic portal gradient], [liver biopsy] AND [liver stiffness] AND [Viral hepatitis].

Selection Criteria

The following filters further narrowed the selection: clinical study, clinical trial, controlled clinical trial, multicenter study, observational study, randomized control trial, studies published within the last 15 years, studies published in the English language, and studies involving humans.

Inclusion criteria: Studies published from 2005 to 2018 were considered for inclusion. The study population included human patients, both male and female, adults, and children with cirrhosis and hepatitis C, and patients who had undergone measurements of liver stiffness and HVPG to predict outcomes or had the gold standard of liver biopsy in comparison. Studies describing the progression of cirrhosis/hepatic fibrosis and those assessing the association of hepatitis C-related injury with comorbidities, including HIV, portal hypertension, and esophageal varices, were considered for inclusion. Prospective cohorts accounted for a large portion of the research criteria as measuring the progression of cirrhosis is a lengthy process that requires time to produce significant results.

Exclusion criteria: Animal studies, systematic reviews, meta-analyses, and articles older than 2005 were excluded.

Results

In a study comparing liver stiffness measurements (LSMs) to liver biopsy, Ziol et al. (2004) [4] assessed the liver stiffness of patients using ultrasound TE with the current gold standard of fibrosis staging using a biopsy sample. The goal was to investigate the efficacy of using the non-invasive LSM to evaluate liver fibrosis in 327 chronic hepatitis C patients in a prospective multicenter study. Of note, 65% (214) of the included patients were from the Jean Verdier Hospital in Bondy, France. Of the 327 patients with no ascites, only 251 patients were included due to specific criteria that met reliable stiffness measurements and a suitable biopsy for fibrosis stage assessment. All patients who underwent a liver biopsy and had a positive HCV RNA in serum from November 2002 to September 2003 were included in the study. LSM was performed six months after LB. LSM had a success rate of 60% or greater and was calculated by the number of successful acquisitions over the total number of acquisitions. The study demonstrated that liver stiffness was significantly different between patients according to their fibrosis stages (p < 0.0001) with a positive correlation of 0.55. Fibrosis staging was agreed upon by two pathologists and analyzed using a kappa coefficient value of 0.90 (95% CI = 0.77-1.02). Integration of METAVIR into the study allowed for proper fibrosis staging, progression activity, and fatty changes. When liver stiffness was evaluated next to activity or steatosis, there were significant differences with p-values <0.0001 and 0.0008, respectively. However, when all three variables were analyzed, only fibrosis was significantly correlated with liver stiffness.

Kim et al. (2016) [5] collected clinical data from a retrospective cohort of 97 cirrhotic patients between 2009 and 2012. Using the CTP and MELD scores, the long-term prognosis of disease severity was categorized. These parameters from the scoring systems include portal hypertension, ascites, hepatic encephalopathy, and variceal bleeding. To determine decompensated cirrhosis complications, the HVPG was measured with the optimal cut-off marker at 17 mmHg. Of the 97 patients, 41 were categorized as stage 3 decompensated cirrhosis which was defined as patients with ascites presenting with or without variceal bleeds. An additional 56 patients were categorized as stage 4 which was defined as those with variceal hemorrhage and combined ascites or no ascites. Of the 56 stage 4 patients, 31 had ascites, and 25 did not have ascites. According to the recorded findings from the HVPG, pressures were higher in patients complicated by ascites with a statistically significant p-value of 0.015. All patients were followed up after 24 months to observe mortality outcomes plotted on Kaplan-Meier survival graphs. Of the patients with HVPG measurements above 17 mmHg, there was an association with mortality risk if the patient had ascites versus no ascites compared to the group whose HVPG was measured below 17 mmHg. Further supporting the evidence, there was a significant reduction in mortality at the one- and two-year marks if the HVPG was above or below the 17 mmHg level, as indicated by the following: 1.9% and 11.9% with HVPG <17 mmHg and 16.2% and 29.4% with HVPG >17 mmHg, respectively, and a p-value of 0.015. The study also noted that MELD scores could more accurately predict survival than CTP and HVPG as the score increased with worsening parameters. When MELD scores were compared to HVPG at one and two years, the overall mortality on the area under the receiver operating characteristics curve (AUROC), there were no statistical differences supported by a p-value <0.0001. The primary conclusion was that the HVPG is a useful instrument for predicting long-term mortality for decompensated patients complicated by ascites.

Studies Comparing Liver Stiffness to Hepatic Venous Portal Gradient

Vizzutti et al. (2007) [6] weighed the comparative measurements of liver stiffness using TE and HVPG in 61 patients with chronic HCV disease. Of the 61 patients enrolled in the study, 12 had clinically significant HVPG >10 mmHg and 35 patients had severe HVPG >12 mmHg. Additionally, the 47 cirrhotic patients were categorized as having clinical or severe portal hypertension, 30 of whom had esophageal varices. In conjunction with HVPG, there was a positive correlation with LSM, as determined by an r-value of 0.81 and a p-value of 0.0003 for the entire patient population. Liver stiffness was more apparent if the HVPG was >10 mmHg or >12 mmHg. However, the diagnostic accuracy of detecting clinically significant portal hypertension had a better positive predictive value (PPV) and negative predictive value (NPV) if the LSM cutoff was 13.6 kPa vs. 17.6 kPa. This was supported by a high sensitivity of 97% and a high specificity of 94%, indicating the ability of the LSM test to correctly identify those with clinically significant portal hypertension.

In a prospective cohort study performed over five years, Facciorusso et al. (2018) [7] administered interferon to 70 patients in one arm of the study and non-interferon, direct antiviral drugs to 83 subjects in the other arm of the study. Direct antiviral agents are a class of drugs that include protease inhibitors and nucleotide/nucleoside inhibitors used in preventing specific steps in the replication and entry of HCV. Of the 153 enrolled, 53 were diagnosed with cirrhosis. Additionally, within the arms of the study, 112 patients responded to treatments, and hepatitis C was not detected in the blood 12 weeks after completing the treatments. On liver stiffness testing, kilopascals in the treatment group had a significant reduction from 12.3 kPa to 6.6 kPa by the end of the study, indicating the recession of fibrosis due to treatment. Conversely, those who did not achieve sustained viral response (SVR) to the treatments had a vague insignificant decline in liver stiffness at the end of treatment; however, intrahepatic pressures were elevated after the five-year study duration. Treatment along with using LSM provided better results, and the number of patients with cirrhosis after five years reduced to fewer than 27, followed by less than three in those who were originally diagnosed at the beginning of the prospective study.

Tseng et al. (2018) [8] examined a novel three-dimensional (3D) computed tomography (CT) rendering technology to closely analyze liver and splenic volumes to gauge HVPGs. The goal was to reduce mortality in patients with decompensated cirrhosis complications. The study retrospectively collected data from 77 patients who underwent HVPG testing and endoscopy for variceal grading, followed by CT volume calculations for the liver and spleen. Parameters included total bilirubin, albumin, aspartate aminotransferase, serum creatinine, hemoglobin, platelet count, PT, and aspartate aminotransferase to platelet ratio index (APRI) obtained at the time of admission. Utilizing the IQQA-liver model, cubic centimeters of liver or spleen volumes were constructed to evaluate portal hypertension as these organs have tendencies to grow in advanced stages. Radiological findings for liver volume had a mean of 1,138.81 ± 407.89 cm^3^ (reference range = 984-2,439 cm^3^) and splenic volumes had a mean of 848.73 ± 399.13 cm^3^ (reference range = 107.2-314.5 cm^3^). Endoscopy identified 38 patients with dilation of the submucosal veins continuing along the lesser curvature of the stomach, identified in the study as gastroesophageal varices (GOV) type 1. In total, 22 patients were categorized as having esophageal varices spanning into the fundus portion of the stomach, i.e., GOV type 2. This patient population was found to have average hepatic venous pressures of around 14.25 mmHg. Any clinically significant portal hypertension >10 mmHg produced a sensitivity of 80.36% and specificity of 76.19% with an AUROC of 0.810 (95% CI = 0.705-0.891) with an optimal cut-off of 12.84 mmHg. Importantly, viral cirrhosis was diagnosed in 47 of the 77 enrolled (61.03%) patients, with 44 hepatitis B and three hepatitis C cases. The remaining 30 were due to non-viral factors. This study investigated the differences in the HVPG tool when comparing viral and non-viral statistics. Patients with viral cirrhosis resulted in an AUROC of 0.798 (95% CI = 0.655-0.901) compared to the non-viral cirrhosis AUROC of 0.820 (95% CI = 0.637-0.935).

Hong et al. (2013) [9] selected 59 patients with confirmed cirrhosis who had quantifiable hepatic portal venous pressures taken from February 1, 2009, to February 1, 2010. In this cohort, all were subjected to a TE fibroscan, in which reliable results included a success rate of 60% and an interquartile range of <30%. Comparative analysis used to relate the significant findings between HVPG and LSM resulted in a linear regression plot with a positively significant correlation in the entire population for these variables but were more highly scrutinized as independent variables. As the HVPG increased above 12 mmHg, the correlation to LSM became weaker, r = 0.192, in parallel to the >10 mmHg group which had an r-value of 0.297. This finding can be seen in multiple studies where the efficacy of LSM is reduced as advanced cirrhosis and severe portal hypertension are manifested. This data is supported by a decline in LSM specificity and PPVs as the HVPG increases to >12mmHg.

Mauro et al. (2018) [10] hypothesized that SVR improves survival in post-liver transplant in recurrent hepatitis C patients. Interpretations of SVR for 112 patients were conducted using a comparative analysis of HVPG, LSM, and the Enhanced Liver Fibrosis (ELF) score. Within the cohort, 52 subjects were given interferon (INF)-based therapies and 60 were administered INF-free therapies. The INF group had better outcomes and significantly lower HVPG, LSM, and ELF scores compared to the non-INF group during baseline and follow-up SVR evaluations. When baseline levels were recorded, patients were categorized into a METAVIR fibrosis stage to determine cirrhosis severity. Most importantly, the HVPG pressure in the study significantly reduced at the 12-month follow-up from 8.5 mmHg to 6.0 mmHg, as supported by a p-value <0.001 in the INF-treated subgroup. As determined from the LSM, the fibrotic nature of cirrhosis receded in the 12-month follow-up by 47%. This claim was supported by an AUROC of 0.653 (0.545-0.772), a PPV of 78%, and an NPV of 44% which could correctly classify 55% of these patients.

Lemoine et al. (2008) [3] attempted to confirm at which kilopascal value LSMs were optimal to define the diagnosis for clinically significant portal hypertension in hepatitis C patients. The study included patients who received a transjugular liver biopsy and LSM using fibroscan (TE) from January 2004 and September 2006 with HVPG taken the same day. The study excluded patients who had other non-HCV causes of cirrhosis along with any mixed criteria such as hepatitis C and alcohol, coinfections, or current beta-blocker therapy. At the time of the study, all subjects were staged with class A in line with the CTP grading system. Additionally, to discover a link between LSM and HVPG in cirrhotic patients, TE using fibroscan was conducted in 92 patients with a success rate reliability of 70%, 10 validated acquisitions, and an interquartile range of less than 30 of the median value in kilopascals. According to the data presented, linear regression analysis for the entire patient population demonstrated a moderate positive r-value of 0.53 and a significant p-value <0.0001, which indicated a correlation. This result was seen in multiple studies. When combined with that of HCV patients, the correlation between HVPG and LSM became slightly weaker with a reported r-value of 0.46. Furthermore, AUROC values demonstrated the same weak correlation. The entire population for HVPG to LSM was 0.84 while when narrowed down to the HCV cause of cirrhosis, the AUROC further decreased to 0.76.

Silkauskaite et al. (2009) [11] documented 128 patients with cirrhosis from 2006 to 2008, authenticated by liver biopsy to establish a CTP class of disease severity. Of the 128, 100 were identified as radical CTP class B or C (78.1%) with the majority (44.5%) diagnosed with hepatitis B or C. Additionally, at the time of the study, patients were checked for any esophageal varices, of whom 89 (69.5%) had large varices identified on endoscopy. A significant p-value of 0.007 supported the higher increases in HVPG correlated with larger varices and thus mortality. However, non-bleeds were more often the finding in patients with large varices. Evidence provided upheld their claim that higher hepatic venous portal pressures were in fact associated with patients who tend to bleed.

Awad et al. (2013) [12] conducted a prospective cohort study using 33 children with chronic hepatitis C who were admitted to the pediatric ward at Tanta University Hospital in Egypt. Based on their LSM score in kilopascals, patients were assigned an F score indicating disease severity. The higher the kilopascals, the greater the F score. Under the scrutiny of the pathologists assessing the liver biopsy, patients were given a METAVIR F score labeled as F0 = no fibrosis, F1 = portal fibrosis without septa, F2 = portal fibrosis with few septa, F3 = numerous septa without cirrhosis, and F4 = cirrhosis. According to the LSM values, there were significant deviations in mean F3 and F4 groups in opposition to F0, F1, and F2 groups, as indicated by a p-value of <0.0001. In other words, liver stiffness increased markedly in more severe hepatic fibrosis cases. Further supporting the data in predicting the success of LSM for fibrosis staging, there was a distinction from the F1 stage to the F4 in terms of sensitivity, specificity, PPV, NPV, and accuracy of the values. As the F score increased, the accuracy of LSM improved as did the sensitivity, specificity, PPV, and NPV. These statistical parameters were in line with the fact that as fibrosis advanced to severe and significant stages, the liver had more areas of hardened tissue from chronic inflammation.

Discussion

The results of this literature review support that TE and HVPG are successful in showcasing the progression of cirrhosis to more complicated stages that are highly involved in the complications manifested by severe disease. Even with the limitations of each study, the data supports the use of alternative modalities in patients with all-cause cirrhosis, especially viral causes, which is the foundation of this paper.

The study by Ziol et al. (2004) [4] used experienced pathologists to attenuate any interobserver bias on biopsy samples. In this study, consensus exists among pathologists for criteria and characteristics of fibrosis in biopsy samples. However, it was mentioned that because liver biopsy only samples a small piece, and it is known that cirrhosis takes on a mosaic pattern in liver tissue exhibiting different fibrosis staging, it is challenging to use just one liver biopsy sample to incur speculation of advanced disease. Moreover, supporting the hypothesis of this paper, fibroscan (TE) could investigate a larger area over 100 times the size of the standard liver biopsy. This makes TE valuable regarding patient acceptance and the ability to accurately diagnose. Inversely, the limitations withheld in elastography are clear in advanced stages when portal hypertension increases above 12 mmHg and when the risk of ascites is present. The elastic waves can not travel through fluid or fat and thus present a problem if the patient is morbidly obese or has abdominal fluid distension from loss of osmotic pressure. Therefore, LSM is better suited in the early stages and is crucial for early diagnosis to possibly improve treatment outcomes. Additionally, the success rates of LSM over biopsy were notably better possibly due to fewer failure variables such as obesity rates in France versus the United States. It would be imperative to conduct further studies in these areas to equate any significant differences. Due to young opioid users, the large number of HCV cases in the United States creates another problem in being able to diagnose all cases on liver biopsy alone. This calls for alternative methods to determine prognosis efficiently and accurately without raising the risks involved. Thus, fibroscan is one example of the benefit to these patients.

Kim et al. (2016) [5] concluded that HVPG is useful in the mortality prognosis of patients with ascites; however, the MELD score was more suited to accommodating disease severity changes as the HVPG increased above 12 mmHg and a 17 mmHg cut-off value. Even though the AUROC was 0.687, the authors were hesitant on HVPG predicting mortality as patients typically have portal pressures exceeding 12 mmHg. At the same time, as those presenting with ascites will also have higher portal pressures, the clinical staging for these patients can become blurred when compared to the MELD scoring system which takes these into account and classifies patients more accurately. The main drawback of this study was the limitation to all causes of cirrhosis and the use of strictly non-critically ill patients with decompensated cirrhosis. The study selected patients who were stable and had no active bleeds or alcohol use. The study still has value in terms of using HVPG as a cirrhosis indicator.

Vizzutti et al. (2007) [6] supported the hypothesis of this paper by stating that TE is a safe and respectable alternative for anticipating advancing changes in portal venous pressure due to increasing stiffness. One of the risky complications seen in patients with cirrhosis is the high mortality associated with ruptured esophageal variceal bleeding. Ongoing chronic modifications to the liver histology led to increases in the intrasinusoidal portal pressure and the formation of these varices >10 mmHg. Reliable data can be formulated when LSM is combined with HVPG. However, the limitation of the study was the consideration of using upper GI endoscopy to rule out a bleeding risk officially. Therefore, multiple approaches are necessary as patients develop more decompensated complications.

Facciorusso et al. (2018) [7] outlined the antiviral responses in relation to liver stiffness over a period of five years. They observed that once patients did not have detectable HCV RNA in serum, the level of fibrosis picked up by fibroscan (TE) showed decreasing kilopascals. This detection was an essential aspect of this study because they also reported a decline in cirrhosis in almost all of their patients with cirrhosis. Hence, as more patients respond to treatment, the success rates of LSM and HVPG to have efficacious diagnoses become more significant.

Tseng et al. (2018) [8] sought the advantage of 3D CT radiographical imaging as a non-invasive tool to gauge HVPG through increasing liver and spleen volumes. Because of its availability in most hospitals and clinics, this provides a potential alternative to disease staging. In viral cirrhosis, the liver demonstrated smaller volumes implying atrophy of the hepatocytes. Conversely, in alcoholic liver disease, an enlargement in the size of hepatocytes (1,600-1,700 µm^2^) occurs before an increase in the intrahepatic and portal pressures. These findings likely point to the necrotic tropism of HCV to hepatocytes versus alcohol being a mitochondrial toxin. Emphasizing the point made previously in the study by Ziol et al., TE can provide encouraging results yet be constrained by the patient’s body mass index (BMI) and whether they have ascites. This was no different in the study by Tseng et al..

Hong et al. (2013) [9] focused their study much in the same way as others mentioned in the article. LSM is highly related to HVPGs, as concluded in multiple studies on the subject. A strong positive correlation was reported based on the AUROC of 0.877. Although HVPG is regarded as a safe invasive method, the authors agreed that more methods should be utilized as cirrhosis is a complex disease with multiple factors to consider. Based on HVPG levels, LSM diagnosed clinically significant portal hypertension with cut-off values of 13.6 kPa to 34.9 kPa. This demonstrates how variable the intrahepatic pressures can be across multiple patients with different sets of parameters. They concluded this to be due to most of their study population having alcohol-induced cirrhosis and not virus-induced cirrhosis. Therefore, the pressures reported in this study are higher than those reported in other studies that had lower cut-off values. Another limitation of the study was the lack of liver biopsies to confirm the current gold standard. This could weaken the hypothesis of this study as no comparison was done.

Mauro et al. (2018) [10] concluded that when liver transplant patients secured undetectable viral levels in serum, the regression from a fibrotic state increased a year after the treatment. As determined by LSM and HVPG taken post-SVR, the study supported the use of these methods as important follow-up tests after patients have been treated. In the 52 patients treated with INF vs. INF-free (direct antiviral agents) therapies, there was significant fibrosis reduction revealed by decreases of more than one METAVIR stage. This is the current gold standard for assessing regression. However, this was uniquely seen in single-variable analysis. When evaluated with multiple variables such as baseline HVPG, the presence of fibrous septa, and CSPH, the fibrosis regression was insignificant because the authors reasonably indicated differences in baseline levels for the two treatment arms of the study. This study was limited to the time in which the data was collected. Longer studies should be performed as chronic inflammatory states are not restricted by time and can continue to progress even after trials are concluded. The main limitation of the Mauro et al. study was sampling bias and their definitions of fibrosis regression. Larger samples are needed to accurately depict progressing cirrhosis and be confined to a specific method of defining regression instead of several different ways as the study contrasted.

The study by Lemoine et al. (2008) [3] showed similar results to the previously mentioned Vizzutti et al. (2007) [6] study in regard to the relationship between HVPG and LSM. A major difference between HVPG and LSM was the AUROC values calculated. The authors noted that this finding could have been due to inclusion and exclusion criteria for their methods. This study included multiple causes of cirrhosis which provided a framework for differences in liver architecture that occur with alcohol versus viral causes. This was also seen in the Tseng et al. study which described viral atrophy and alcoholic hypertrophy changes that could skew the LSM results. Of note, Lemoine et al. (2008) emphasized the need for elastography because it has outstanding results in diagnosing cirrhosis at the same time estimating portal hypertension while being pain-free and cost-effective. Patients are more willing to undergo this type of testing over liver biopsy which causes pain and bleeding.

Silkauskaite et al. (2009) [11] demonstrated that hepatic venous pressures were higher in patients with CTP class B and C and were more likely to develop esophageal variceal bleeding. However, it was not clear if there were differences in HVPG in those who bled with large varices to those who did not, citing it as a confounding variable. It would be of great use to further investigate this using confidence intervals and r values to pinpoint whether or not a confounding variable is present in the study. That said, HVPG was reported to be higher in bleeders over those who did not bleed.

Awad et al. (2013) [12] supported the notion that TE could be used to replace liver biopsy in many patients. Because the incidence rate of HCV is on the rise in the United States, this would save not only potential harm to patients but their costs as well. As predicted, liver stiffness by TE increased with the increased staging of fibrosis, further encouraging the implementation of this non-invasive device as a possible future gold standard. The authors were aware of the limitations within the study that should be taken into consideration to profile future subjects for better results. These included using larger sample sizes and more biopsy samples to accurately allow any definitive study conclusions. Additionally, assessing the patient profile for BMI, whether ascites was present, and other hemodynamic studies including albumin, PT/INR, and liver enzymes would indicate disease severity. This study on children with chronic HCV had comparable fibroscan results seen in other studies with adults judged by agreeable pathologists. As the authors mentioned, the stiffness of liver tissue largely depends on the structure of collagenous material and the organization the elastic waves propagate as cirrhosis has a heterogeneous pattern. This study, and many similar studies, highlight the direction medicine should go in carrying out HCV prognosis for those who develop a chronic condition and have complications or potentially mortality-related events.

## Conclusions

In summary, according to the CDC, hepatitis C cases are predicted to substantially increase over the next 10 years. Because of this public health threat, liver biopsy is becoming a questionable procedure as more patients require diagnosis and fibrosis staging. Its drawbacks result in an unfavorable approach for patients who may require a larger sample to assess cirrhosis, as well as in whom the non-invasive LSM, TE, may provide a more beneficial picture of overall advanced fibrosis or cirrhosis in patients with chronic hepatitis C. Based on the collective information, the use of less invasive modalities such as HVPG and TE with fibroscan is better for diagnosing cirrhosis in hepatitis C patients despite their limitations. A limitation may be the choice of hepatic venous pressure measurement as a non-invasive tool, as it is invasive and not readily available in routine clinical care, unlike TE using fibroscan, which is truly non-invasive and is more readily available for routine practice. Ultimately, more studies are needed to produce higher-quality results across several demographics.
